# Atypical COVID-19: Preventing transmission from unexpected cases

**DOI:** 10.1017/ice.2020.419

**Published:** 2020-08-13

**Authors:** Angela Chow, Htet Lin Htun, Win Mar Kyaw, Hou Ang, Glenn Tan, Huei Nuo Tan, Li Wearn Koh, Bernard Yu-Hor Thong, Brenda Ang

**Affiliations:** 1 Department of Clinical Epidemiology, Office of Clinical Epidemiology, Analytics, and Knowledge (OCEAN), Tan Tock Seng Hospital, Singapore; 2 Lee Kong Chian School of Medicine, Nanyang Technological University, Singapore; 3 Department of Emergency Medicine, Tan Tock Seng Hospital, Singapore; 4 Department of General Surgery, Tan Tock Seng Hospital, Singapore; 5 Department of Geriatric Medicine, Tan Tock Seng Hospital, Singapore; 6 Department of Rheumatology, Allergy, and Immunology, Tan Tock Seng Hospital, Singapore; 7 Department of Infectious Disease, Tan Tock Seng Hospital, Singapore

*To the Editor*—Much is still being discovered about coronavirus disease 2019 (COVID-19). Although originally described as viral pneumonia, reports of nonrespiratory manifestations of COVID-19 are increasing, including gastrointestinal, ocular, cardiac, and neurologic presentations.^1–3^ Hospitals restrict visitors with acute respiratory infections (ARIs), isolate and test all ARI patients for severe acute respiratory coronavirus virus 2 (SARS-CoV-2), and provide staff with full personal protective equipment when managing ARI patients.^4^ Concerns about how to titrate infection control measures for asymptomatic, presymptomatic, and atypical infections^5^ are heightened by fears of subsequent waves of COVID-19 from easing lockdowns. Transmission most likely occurs via droplets from infected individuals with respiratory symptoms, but it remains unclear whether SARS-CoV-2 is just as transmissible by patients who do not have respiratory symptoms.

Since January 23, 2020, the 1,600-bed Tan Tock Seng Hospital, co-located with the 330-bed National Centre for Infectious Diseases (NCID), has managed >9,000 COVID-19 patients.^6^ Contact tracing was immediately performed for every COVID-19 patient not managed at designated COVID-19 areas (fever zones) in the emergency department or clinic, or not pre-emptively admitted to an airborne infection isolation room (AIIR). In fever zones, all staff donned N95 respirators, gowns, gloves, and eye protection, while patients and visitors wore surgical masks. In non–fever zones, staff, patients, and visitors wore surgical masks and observed standard precautions.

Between January 23, 2020, and July 25, 2020, the hospital managed 5 COVID-19 patients without ARI symptoms who presented with gastrointestinal symptoms (n = 2), upper-limb swelling (n = 1), fever and muscle pain (n = 1), and anxiety symptoms (n = 1) (Table 1).

**Table 1.** Characteristics of Patients and the Exposed Contacts

Characteristics	Patient 1	Patient 2	Patient 3	Patient 4	Patient 5
Age, y	68	80	55	90	42
Sex	Female	Male	Male	Female	Male
Race	Chinese	Chinese	Chinese	Chinese	Chinese
Pre-existing conditions	Nasal tip basal cell carcinoma	Eosinophilic granulomatosis with polyangiitis, gastric adenocarcinoma, chronic renal impairment, active hepatitis B infection	None	Alzheimer’s disease, hypertension, hyperlipidemia, diabetes mellitus, paroxysmal atrial fibrillation	Hyperlipidaemia, gastroesophageal reflux
Presenting symptoms	Nausea and abdominal pain	Persistent middle-finger tenderness, and upper-limb swelling	Fever, muscle pain, rash, and nausea	Vomiting, lethargy, and foul-smelling urine	Chest pain and palpitations
Admitting diagnosis	Nausea likely secondary to gall stones	Infective tenosynovitis	Pulmonary tuberculosis, Dengue fever	Pneumonia, functional decline	Atypical chest pain
Radiologic evidence of pneumonia	Yes	Yes	Yes	Yes	No
Managed at the outset at COVID-19 designated areas in the ED/clinic	No	No	No	No	Yes
Time to isolation at COVID-19 designated areas in ED/clinic, h	NA	NA	3	3	NA
Admitted from ED/clinic directly to a single room	No	No	Yes	Yes	Yes, but subsequently de-isolated
Time in an open ward, h	48	44	NA	NA	147
Length of hospitalization, d	8	13	11	6	9
Exposed contacts, no.	92	33	35	20	67
**Contacts with unprotected exposure, no.**					
Patients	34	4	0	0	9
Visitors	6	0	0	0	0
Caregivers	2	0	0	0	0
Staff	1	0	0	0	0
**Contacts with protected exposure, no.** ^ab^					
Patients	0	0	0	1^a^	0
Visitors	0	0	0	0	10^a^
Caregivers	0	0	0	0	0
Staff	49 (42^a^ and 7^b^)	29 (28^a^ and 1^b^)	35 (15^a^ and 20^b^)	19 (13^a^ and 6^b^)	48^a^
Symptomatic contacts screened for SARS-CoV-2, no.	11	3	5	0	0
Asymptomatic contacts screened for SARS-CoV-2, no.	0	0	0	0	48
Contacts confirmed with COVID-19, no.	0	0	0	0	0

Note. ED, emergency department.

a Surgical mask.

b N95 respirator, gown, gloves, eye protection.

## Patient 1

A 68-year-old woman presented to the emergency department non–fever zone on February 27, 2020, with a 2-week history of epigastric pain and nausea without fever or respiratory symptoms. She was admitted to a 5-bed cubicle in a general ward. On February 28, an abdominal/pelvic computed tomography scan revealed ground-glass changes in her bilateral lower lungs. She was immediately transferred to a single-bed room. SARS-CoV-2 was detected on the second nasopharyngeal swab taken on March 1. She was transferred to an AIIR, recovered uneventfully, and was discharged on March 5.

We identified 92 contacts (Table 1). Among them, 8 were inpatients who were moved to single-bed rooms. One staff member who had unprotected close contact developed ARI symptoms on March 1 and was admitted to the NCID, but 2 nasopharyngeal swabs collected 24 hours apart were negative for SARS-CoV-2. Moreover, 3 close patient contacts and a caregiver developed ARI symptoms, as well as another close patient contact whose chest x-ray showed worsening of air-space changes. They were screened for SARS-CoV-2, and the virus was not detected in ≥2 nasopharyngeal swabs taken 24 hours apart in these contacts. Also, 4 casual patient contacts and a staff member with protected exposure who became symptomatic also screened negative for SARS-CoV-2.

## Patient 2

On March 20, 2020, an 80-year-old Chinese man with multiple comorbidities was admitted to a 5-bed cubicle from the rheumatology clinic for persistent left middle-finger tenderness and upper-limb swelling. A chest x-ray showed resolving right-lower-zone pneumonia. He had a fever (38.2°C) on March 22 and was transferred to a single-bed room. SARS-CoV-2 was detected on the nasal swab taken on the same day. He was transferred to an AIIR and was discharged on April 1 after recovering. He never developed respiratory symptoms.

We identified 33 contacts. All patient contacts remained asymptomatic, and 3 symptomatic staff contacts tested negative for SARS-CoV-2.

## Patient 3

A 55-year-old Chinese man presented to the emergency department non–fever zone on March 24, 2020, with a 3-week history of intermittent fever, rash, nausea, and lethargy. The chest x-ray suggested an atypical infection or pulmonary tuberculosis. A Dengue Duo test showed IgM and IgG positivity. He was admitted to a single-bed room. SARS-CoV-2 was detected in the nasal swab taken on the same day, and he was transferred to an AIIR. Tuberculosis was ruled out by negative acid-fast bacilli smears and cultures. He did not develop any respiratory symptoms and was discharged on April 3 after recovering.

We identified 35 contacts, all staff. Among them, 5 developed symptoms but tested negative for SARS-CoV-2.

## Patient 4

On April 20, 2020, a 90-year-old Chinese-woman with multiple comorbidities presented to the emergency department non–fever zone with a 1-day history of vomiting, lethargy, and foul-smelling urine. She had no fever or respiratory symptoms, but a chest x-ray revealed left-lower-zone air-space opacities and pleural effusion. She was admitted to a single-bed room. SARS-CoV-2 was detected on the second sample. She was immediately transferred to an AIIR and was discharged on April 25 after recovering. All 20 contacts remained asymptomatic until 14 days after exposure.

## Patient 5

A 42-year-old Chinese man, a resident of a dormitory with COVID-19 transmission, presented with chest pain and palpitations on June 16, 2020. He was admitted to an AIIR but was transferred to a 5-bed cubicle for treatment of an anxiety disorder after testing negative for SARS-CoV-2. The day after his discharge on June 22, he was screened for SARS-CoV-2 in preparation for China travel and tested positive.

Of 3 in-hospital patient contacts, 1 was detected with SARS-CoV-2 on June 25. This patient had been admitted for congestive cardiac failure and suspected pneumonia. This patient contact and patient 5 had reactive SARS-CoV-2 serological tests taken on June 27 and 28, respectively; thus, it was improbable that the patient contact acquired COVID-19 from patient 5 while receiving care in the same cubicle.

In conclusion, we have reported 5 patients not initially suspected with COVID-19 and thus not managed at COVID-19–designated areas or AIIRs. In total, 247 contacts were identified from the hospital’s patient and visitor registration systems, staff rosters, electronic medical records, and closed-circuit television. Also, 56 contacts with unprotected exposure were quarantined or placed under phone surveillance for 14 days after exposure. Finally, 19 symptomatic contacts and 48 asymptomatic staff contacts were tested for SARS-CoV-2; none were positive for the virus.

Because the same vigilance for ARI patients cannot realistically be implemented for the smaller proportion of patients with atypical symptoms, the following measures are crucial for preventing nosocomial transmission of SARS-CoV-2: a robust hospital system with risk-based personal protective equipment, staff sickness surveillance,^7^ and rapid identification of COVID-19 patients with immediate contact tracing and management.
